# Improvement of pulmonary edema and respiratory status after transcatheter PDA closure in the smallest and most premature infants

**DOI:** 10.3389/fped.2025.1472431

**Published:** 2025-04-24

**Authors:** Ahmad Chmaisse, Kaitlin E. Swanson, Michael M. Ross, Matthew L. Cooper, Diane E. Lorant, Ryan D. Alexy

**Affiliations:** ^1^ Division of Cardiology, Riley Hospital for Children, Indiana University School of Medicine, Indianapolis, IN, United States; ^2^ Department of Radiology, Riley Hospital for Children, Indiana University School of Medicine, Indianapolis, IN, United States; ^3^ Division of Neonatology, Riley Hospital for Children, Indiana University School of Medicine, Indianapolis, IN, United States

**Keywords:** interventional pediatric cardiology, transcatheter PDA closure, preterm infants, respiratory status, patent ductus arteriosus

## Abstract

**Objective:**

We evaluated the effect of transcatheter PDA closure (TCDC) on pulmonary edema by chest x-ray and respiratory status in preterm infants and identified factors contributing to clinical improvement.

**Study design:**

A retrospective review of TCDC in 68 premature infants from January 2017 to June 2021. Chest x-rays were reviewed to assess pulmonary edema. Multiple clinical characteristics were also evaluated.

**Results:**

40% of patients weaned respiratory support. x-ray haziness change was not significantly different between groups (*p* = 0.086), however trended toward significance. 59% had decreased haziness and 16% had a marked decrease. Smaller, younger infants were more likely to wean support and have improved edema.

**Conclusion:**

Chest x-ray haziness improved after TCDC, with smaller infants and earlier closure having more improvement. Infants with lung disease had less noticeable improved edema, indicating the difficulty to assess the hemodynamic significance of their PDA prior to closure. Further studies are needed to identify which neonates benefit most from TCDC.

## Introduction

During gestation, oxygenated blood from the placenta reaches the systemic circulation via shunts that are present *in utero* and normally close shortly after delivery. One of these shunts is the ductus arteriosus, which diverts blood away from fluid-filled lungs (1). In full-term infants, postnatal changes in hemodynamics and vasoregulatory mediators typically result in ductal closure within 72 h (2–4). In preterm infants, ductal closure is often delayed due to structural immaturity and inadequate constrictive responses, with the likelihood of patency inversely proportional to gestational age (3). Studies have shown that the likelihood of ductal patency after birth is inversely proportional to the gestational age and weight at birth (5–8).

“Ductal steal” from a patent ductus arteriosus (PDA) can lead to pulmonary overcirculation, edema, and organ hypoperfusion, with reported adverse outcomes such as pulmonary hypertension, bronchopulmonary dysplasia (BPD), other end-organ injuries, and increased mortality (3, 8–17). A few scoring systems have attempted to incorporate clinical and echocardiographic findings to assess for hemodynamically significant PDA's (hs-PDA's), with a noted one being the McNamara score (18).

Management options for PDAs include pharmacologic, interventional, and surgical approaches. NSAIDs, such as ibuprofen, remain the first-line treatment for PDA closure (19). Recently, early transcatheter PDA closure (TCDC) has emerged as a viable option, especially for infants who fail medical management. A 2022 study (20) using data from the Pediatric Health Information System (2016–2020) reported that TCDC in neonates and infants is associated with lower mortality (0% vs. 1.7%) and shorter hospital stays compared to surgical ligation. Similarly, a 2024 study (21) from the Vermont Oxford Network database (2018–2022) found that very low birth weight infants undergoing TCDC had a slightly shorter median hospital stay (128 vs. 132 days) compared to surgical closure. A non-randomized clinical trial (22) on the Amplatzer Piccolo Occluder in patients ≥700 g showed a 95.5% implant success rate, 99.4% achievement of the primary effectiveness endpoint, and a 2.1% rate of primary safety endpoint events. Despite these promising findings indicating TCDC as a viable and safe option with minimal adverse outcomes, most available data are retrospective, with limited research on optimal timing and short- and long-term outcomes, particularly regarding pulmonary and end-organ function in preterm infants (3, 23).

We routinely perform TCDC in premature infants with a hsPDA at our institution and noticed a small subset of these patients have significantly improved appearance of their chest x-ray (CXR) immediately after TCDC. The purpose of this study is to evaluate the effect of TCDC in premature infants on pulmonary edema and respiratory status, and more specifically to characterize the subset of patients who have significantly improved CXRs immediately after TCDC. Better characterization of the infants in whom the CXR and clinical respiratory status benefit the most from TCDC will help guide which infants should be referred for ductal closure and optimal timing of ductal closure.

## Material and methods

### Data collection and baseline characteristics

Data was collected through a retrospective chart review after obtaining approval from the Indiana University Institutional Review Board (IRB) with waiver of informed consent. All transcatheter PDA-closure cases done at Riley Hospital for Children were identified using procedural billing codes between January 2017 and June 2021. These cases were reviewed, and only inpatients from the NICU born at an age less than 37 weeks gestation and less than 6 months old at the time of closure were included. Patients who required surgical PDA-closure after failed transcatheter intervention were excluded.

We stratified the patients into different categories based on their gestational age (extremely preterm, very preterm, and moderate-late preterm), birth weight (extremely low birth weight, very low birth weight, and low birth weight), and weight at PDA closure. We also included other characteristics such as the use of surfactant therapy at birth, comorbid conditions, prior steroid therapy, prior inotrope requirements, prior diuretic requirements, and respiratory support on the day prior to PDA closure. The Bronchopulmonary Dysplasia (BPD)/Chronic Lung Disease (CLD) criteria used was based on the 2019 classification, which requires a corrected gestational age greater than 36 weeks (24).

### Hemodynamic significance of the PDA

At Riley Hospital for Children, the decision to proceed with transcatheter PDA closure in preterm infants is largely driven by the PDA's hemodynamic significance (hs), determined by the Indiana University (IU) Modified McNamara Scale. This score is a modified version of the original McNamara Score (18). The IU-Modified McNamara Scale (Table 1) is calculated by adding the “Clinical” and “Echocardiographic” scores, with the latter determined by one of the pediatric cardiologists at our institution for this study.

**Table 1:** IU-modified mcNamara scale.

IU-modified McNamara scale	
Clinical score	
Points	Clinical appearance
1	Asymptomatic
2	Mild: FiO2 < 0.3 and NCPAP, MV, or NC > 1 lpm
3	Moderate: FiO2 0.30–0.60 and NCPAP, MV, or NC > 1 lpm OR hypotension requiring a single vasopressor
4	Severe: FiO2 > 0.6 AND MV OR hypotension requiring more than 1 vasopressor
Echocardiographic score	
Points	Echocardiographic findings
1	Continuous flow and increasing velocity flow into the branch PAs: <0.15 m/s in diastole in LPA
2	Small PDA, Continuous flow and increasing velocity flow into the branch PAs: >0.4 m/s in diastole in LPA
3	Moderate PDA, Diastolic flow reversal in the descending aorta below the level of the PDA
4	Large PDA, A dilated LA (typically 2 times larger the aorta in PLAX view)
5	Large PDA, LV dilation

### Chest x-ray (CXR) scoring

To assess the effect of transcatheter PDA closure on pulmonary edema in the immediate post-procedure period, pre-procedure CXR's and post-proc CXR's were reviewed. Considering the dynamic clinical status of premature infants in the NICU, neonates' increased risk for fluid overload with its possible effect on radiographic findings of lung edema, and the decreased frequency of CXR's done at >2 days post-proc, this study looked at the post-proc CXRs that were done only within two days after the procedure. Moreover, we recognize that CXR haziness can reflect various underlying conditions, including pulmonary edema, BPD-associated parenchymal changes, atelectasis, and infection-related consolidations. Therefore, we chose to compare CXRs taken immediately before and after the procedure, hypothesizing that any observed changes in haziness would primarily indicate alterations in pulmonary edema. CXRs were read and scored by 4 independent scorers (2 cardiologists, 1 neonatologist, and 1 radiologist) using the Radiographic Assessment of Lung Edema (RALE) score, initially described by Warren et al. (25) and validated in subsequent studies (26, 27). To calculate the RALE score, the rater scored the haziness in each quadrant of the CXR before adding all 4 values. The rater assigns a consolidation score (C), which assesses the extent of alveolar opacities, as well as a density score (D), which assesses the density of alveolar opacities, and then multiplies the consolidation score by the density score (CxD). The scores from the four quadrants were then added together to get the final total RALE score.

In our study, the four raters evaluated the pre-procedure (pre-proc) CXRs as well as all the post-proc CXRs. To assess the change in haziness between these two periods, we compared the average of the four raters' scores for the pre-proc CXR with the raters' average for the last CXR done within a two-day post-proc period. This time period was chosen given the infrequency of CXRs done after that time, as well as the possibility of other factors affecting the presence of pulmonary edema in the NICU population as described above.

### Respiratory support changes

Tracking changes in respiratory support presented challenges due to frequent fluctuations in FiO2 and other respiratory machine settings, as well as the diverse range of support modalities used before and after the intervention. Initial attempts to analyze specific changes proved overly complex and did not yield meaningful results. Therefore, we focused on assessing the ability to transition to a respiratory support machine that provides less support. This approach was chosen to offer a more comprehensive understanding of respiratory support changes, irrespective of the initial modality.

### Intra-rater and inter-rater reliability

For the intra-rater reliability, the scorers reassessed forty-nine CXRs two months after their initial CXR read. The inter-rater reliability was assessed for the pre-proc CXR and the first three post-proc CXRs.

### Statistical methods

Intra-rater repeatability was assessed by paired *t*-tests and by the intraclass correlation coefficient (ICC). Original and repeated measurements were summarized by mean, standard deviation (SD), median, minimum, and maximum. Differences were calculated for each subject and a paired *t*-test was used to test the hypothesis of no difference. The ICC measured the proportion of total variance coming from between subject variation. One minus the ICC is the proportion of total variance from repeated measurements. Inter-rater reliability was assessed by the ICC and by Bland-Altman plots of differences vs. mean ratings.

Patient characteristics and pre-closure haziness scores, last post-proc haziness scores, and change in haziness scores were summarized by count and percentage for categorical variable and by mean, standard deviation, median, minimum and maximum for continuous variables. Clinical status measures were summarized by count and percentage for day 7 post-proc. Patient characteristics were summarized by clinical status and were tested for differences in distributions by means of Fisher's exact tests and by Wilcoxon rank-sum tests.

Cumulative logistic regression models were used to assess associations between the ordinal outcomes of decreasing support on day 7 and patient characteristics. These estimates are interpreted as the odds of having less support relative to greater support. For the respiratory support data, the order was increased < maintained < decreased. The model assumes the odds are proportional regardless of which levels of the outcome are being compared.

Linear models were used to assess associations between change in haziness and patient characteristics.

A decrease in haziness of at least 16 was used to define groups, and patient characteristics were compared between groups by means of chi-square and Fisher's exact tests.

## Results

### Baseline characteristics

The final number of patients included in the study was 68. Baseline characteristics for our patient population are presented in Table 2. Prior to closure, 74% of patients had a hsPDA. PDA devices used included the Amplatzer Piccolo Occluder (52.2% of patients), Medtronic Microvascular Plug (31.8%), Covidien Microvascular Plug (8.7%), Amplatzer Vascular Plug (1.5%), and Amplatzer Vascular Plug II (5.8%).

**Table 2:** Baseline patient characteristics.

Patient characteristics and distribution by category		*N* (%)
Extent of prematurity	32–37 weeks GA (Moderate-Late Preterm)	1 (1.5)
	28–32 weeks GA (Very Preterm)	8 (11.8)
	<28 weeks GA (Extremely Preterm)	59 (86.8)
Birth Weight	<1,000 (ELBW)	54 (79.4)
	1,000–1,500 (VLBW)	13 (19.1)
	1,500–2,500 (LBW)	1 (1.5)
Weight at PDA Closure	<1,000	5 (7.4)
	1,000–1,500	28 (41.2)
	1,500–2,500	16 (23.5)
	2,500–4,000	13 (19.1)
	>4,000	6 (8.8)
Age at PDA Closure (Months)	*N*	68
	Mean ± SD	1.9 ± 1.2
	Median (Min, Max)	2.0 (1.0, 6.0)
Surfactant Therapy (ST)	No	7 (10.4)
	Yes	61 (89.7)
Steroids	No Steroids	43 (63.2)
	Decadron	12 (17.6)
	Inhalation	10 (14.7)
	Inhalation + Decadron	3 (4.4)
CLD Diagnosis	I	1 (1.5)
	II	11 (16.2)
	III	11 (16.2)
	N/A	45 (66.2)
Indexed Size of PDA/BSA (mm/m2)	*N*	68
	Mean ± SD	23.3 ± 9.1
	Median (Min, Max)	23.7 (4.3, 45.6)
Other Significant L-R Shunts	No	66 (97.1)
	Yes	2 (2.9)
Echocardiographic Evidence of Pulmonary Hypertension	No	37 (54.4)
	Yes	31 (45.6)
IU Echocardiographic McNamara Score	1	5 (7.4)
	3	10 (14.7)
	4	6 (8.8)
	5	47 (69.1)
IU Clinical McNamara Score	2	27 (39.7)
	3	39 (57.4)
	4	2 (2.9)
Total McNamara Score	N	68
	Mean ± SD	7.0 ± 1.2
	Median (Min, Max)	7.0 (4.0, 9.0)
Total McNamara Score ≥7	No	18 (26.5)
	Yes	50 (73.5)

### Haziness change in immediate post-procedure period

A total of 183 CXRs were read and scored. Seven patients were excluded from the study because they did not have a pre-procedure CXR within 4 days of the ductal closure. The haziness change, as per the RALE score, between the pre-proc CXR and the most recent post-proc CXR show a mean change of −2.7 ± 12.2. Mean haziness change was not significantly different from zero with a *p*-value of 0.086. There was a decrease in haziness in 36 (59.0%) of patients, and 10 patients (16.4%) had a decrease of at least 16 which was considered a significant decrease (Table 3). As previously noted, with otherwise stable respiratory and clinical status in the days surrounding the catheterization intervention, these changes were thought to likely reflect variations in pulmonary edema on CXR, the primary outcome of our study.

**Table 3:** Mean haziness change.

Haziness assessment			*p*-value
Mean Haziness Pre-procedure	*N*	61	
	Mean ± SD	31.6 ± 9.7	
	Median (Min, Max)	31.0 (14.0, 48.0)	
Mean Haziness Post-procedure (last chest x-ray)	*N*	61	
	Mean ± SD	28.9 ± 9.9	
	Median (Min, Max)	30.3 (6.5, 48.0)	
Change in Mean Haziness	*N*	61	0.0860
	Mean ± SD	−2.7 ± 12.2	
	Median (Min, Max)	−3.0 (−32.8, 27.0)	
Decrease in Haziness		36 (59.02%)	
Decrease in Haziness of at least 16		10 (16.39%)	

### Relationship between baseline patient characteristics and haziness change

Infants born more prematurely showed a greater improvement in RALE scores, with an average change of 5.2 points lower (indicating less haziness) compared to older infants (Table 4). Older and larger infants tended to exhibit less improvement in RALE scores than their younger and smaller counterparts. Infants who received steroid inhalational therapy had higher RALE scores after the intervention than those who did not. Infants with a larger indexed PDA/BSA, a higher Echo McNamara score, more advanced CLD, or a higher total McNamara score showed greater improvements in RALE scores. Infants with a hsPDA (score ≥7) had an average RALE score improvement that was 8 points lower (less hazy) than those without an hsPDA, with this being the only statistically significant association.

**Table 4:** Haziness change association with patient characteristics.

Patient characteristics and distribution by category		Estimate (std error)	95% confidence interval	*p*-value
Extent of prematurity (compared to very preterm)	Extremely Preterm	−5.162 (5.268)	(−15.703, 5.378)	0.3311
Birth Weight Category (compared to <1,000 g)	1,000–1,500 g	0.262 (4.113)	(−7.968, 8.493)	0.9494
Weight at PDA Closure Category (compared to <1,000 g)	1,000–1,500 g	−1.113 (5.957)	(−13.045, 10.820)	0.8525
	1,500–2,500 g	1.931 (6.456)	(−11.003, 14.864)	0.7660
	2,500–4,000 g	6.927 (6.617)	(−6.329, 20.183)	0.2997
	>4,000 g	4.763 (8.230)	(−11.725, 21.250)	0.5651
Age at PDA Closure (*x* in months)	*x* + 1	1.847 (1.432)	(−1.019, 4.712)	0.2022
Steroids (compared to no steroids)	Decadron	−0.658 (4.038)	(−8.743, 7.427)	0.8711
	Inhalation	8.498 (4.743)	(−1.000, 17.996)	0.0785
	Inhalation + Decadron	2.425 (7.312)	(−12.217, 17.068)	0.7413
CLD Diagnosis (compared to Class II)	III	−4.875 (3.041)	(−11.291, 1.541)	0.1274
Indexed Size of PDA/BSA (mm/m^2^)	*x* + 1	−0.322 (0.178)	(−0.677, 0.034)	0.0754
Echo McNamara Score (as *x* increases by 1)	*x* + 1	−2.051 (1.611)	(−5.275, 1.172)	0.2079
pHTN (compared to not present)	Yes	4.779 (3.122)	(−1.469, 11.027)	0.1312
Total McNamara Score (as *x* increases by 1)	*x* + 1	−2.309 (1.435)	(−5.181, 0.562)	0.1129
Total McNamara Score ≥7 (compared to <7)	Yes	−8.003 (3.613)	(−15.234, −0.773)	**0** **.** **0306**

Bolded values indicate statistical significance at *p* < 0.05.

### Comparison of patient characteristics for those with a haziness decrease of at least 16 points

To look for specific patient characteristics in infants who had a significant improvement in their RALE score after PDA closure (defined as a decrease of at least 16 points, which would represent an improvement of one density point in all 4 quadrants), a comparison of patient characteristics between those with and without a haziness decrease of at least 16 was performed. As shown in Table 5, in patients with a haziness decrease of at least 16, there was a greater proportion of patients who had a weight at PDA closure in the 1,000–<2,000 category, were CLD Diagnosis class 1, were younger on average by about 0.8 months, had a larger Echo McNamara score and were less likely to have pHTN, all of which reached statistical significance (*p* < 0.05).

**Table 5:** Comparison of patient characteristics based on haziness change.

Patient characteristics and distribution by category		Yes 10 (16.4%)	No 51 (83.6%)	*p*-value
Extent of prematurity	Moderate—late/Very Preterm	0 (0.0)	6 (11.8)	0.5769
	Extremely Preterm	10 (100.0)	45 (88.2)	
Birth Weight Category	<1,000	8 (80.0)	42 (82.4)	0.9999
	1,000–2,500	2 (20.0)	9 (17.6)	
Weight at PDA Closure category	<1,000	0 (0.0)	5 (9.8)	**0** **.** **0411**
	1,000–<2,000	10 (100.0)	25 (49.0)	
	2,000–<3,000	0 (0.0)	14 (27.5)	
	3,000+	0 (0.0)	7 (13.7)	
Age at PDA Closure (Months)	Mean ± SD	1.2 ± 0.4	2.0 ± 1.1	**0** **.** **0008**
	Median (Min, Max)	1.0 (1.0, 2.0)	2.0 (1.0, 6.0)	
Surfactant Therapy (ST)	No	1 (10.0)	5 (9.8)	0.9999
	Yes	9 (90.0)	46 (90.2)	
Steroids	No Steroids	8 (80.0)	30 (58.8)	0.3606
	DART	2 (20.0)	10 (19.6)	
	Inhalation/Inhalation + DART	0 (0.0)	11 (21.6)	
CLD Diagnosis	I/NA	10 (100.0)	32 (62.7)	**0** **.** **0235**
	II/III	0 (0.0)	19 (37.3)	
Indexed Size of PDA/BSA (mm/m^2^)	Mean ± SD	26.5 ± 6.6	23.8 ± 9.1	0.3764
	Median (Min, Max)	25.0 (16.4, 35.0)	24.3 (9.2, 45.6)	
Echo McNamara Score	Mean ± SD	4.9 ± 0.3	4.4 ± 1.0	**0** **.** **0060**
	Median (Min, Max)	5.0 (4.0, 5.0)	5.0 (1.0, 5.0)	
pHTN	No	10 (100.0)	24 (47.1)	**0** **.** **0015**
	Yes	0 (0.0)	27 (52.9)	
Total McNamara Score	Mean ± SD	7.5 ± 0.5	7.1 ± 1.2	0.0675
	Median (Min, Max)	7.5 (7.0, 8.0)	7.0 (4.0, 9.0)	
Total McNamara Score ≥7	No	0 (0.0)	14 (27.5)	0.0981
	Yes	10 (100.0)	37 (72.5)	

Bolded values indicate statistical significance at *p* < 0.05.

### Relationship between baseline patient characteristics and respiratory status

We initially compared the clinical status of patients on PPD-1 (post-procedure day) and PPD-7 to their status the day prior to the procedure. To enhance statistical reliability due to the small sample sizes in several patient categories, several patient characteristics, such as prematurity, birth weight, steroid use, and CLD diagnosis, were combined into broader categories. While 10% of patients required increased respiratory support on PPD-1, the trend strongly shifted toward improvement by PPD-7, where 40% of patients required less respiratory support, compared to only 3% who required increased support. The change in diuretic use was less pronounced, with 6% of patients started on or requiring increased diuretics by PPD-7, while 3% had diuretics weaned, and 9% had them completely discontinued. The analysis and results for associations between patient characteristics and outcomes (diuretic requirements and respiratory support changes) using Fisher's exact test and Wilcoxon rank sum tests, are presented in Supplementary Tables A1 and A2.

### Complications

Complications occurred in 9% of patients, including MSSA bacteremia in one patient and one mortality. Inotrope initiation or increase was required in 3% of patients on PPD-1 and in 6% by PPD-7.

### Intra-rater and inter-rater reliability

Looking at intra-rater reliability, repeatability was highest for Rater A. Raters B and C had similar repeatability with 39% of total variation coming from repeated measurements. Rater D had 30% total variation from repeated measurements. Aside from Rater A, the ICC for all raters is considered to be “Good” as per Fleis (28) criteria. The ICC for Rater A is considered to be “Excellent” (Supplementary Table A3).

The Inter-rater reliability was the same for all 4 CXR's assessed. 28% of total variation came from differences between raters. The ICC for the inter-rater reliability had a value of 0.72, which is considered to be “Good” as per Fleis (1986) criteria (Supplementary Table A4).

Of note, an ICC of 0.75 and above is considered to be “Excellent” as per Fleis.

## Discussion

Management of premature infants with a patent ductus arteriosus remains controversial and difficult due to changing practices and lack of robust evidence outlining the indications and benefits. Several studies have been published in the past decade describing the technical success of TCDC in premature infants, with several cohort studies describing a more rapid return to baseline respiratory status after device closure when compared to surgical ligation (20, 29, 30). One of these retrospective studies looked at premature infants undergoing TCDC with The Amplatzer Duct Occluder II Additional Size (ADO II AS, Abbott Vascular, Santa Clara, California, USA) and provided matched patients that underwent surgical ligation. They found faster respiratory improvement after transcatheter closure (30).

Our study was first born from the notion that some patients had marked improvement in chest x-ray appearance after their PDA closure. There is currently no validated assessment tool to quantify pediatric pulmonary edema. Warren, et al. first described the RALE score to evaluate the extent and density of alveolar opacities on chest radiographs in adult patients with acute respiratory distress syndrome (ARDS). There was excellent between-observer agreement for the total RALE score and individual quadrant scores (intra-class correlation coefficient of 0.93) (24). Nearly 60% of our patients showed a decrease in haziness between their pre- and post-procedure CXR. 16% showed a change of at least 16, which is defined as a change of at least one in each quadrant, which was what we deemed to be clinically significant. The expectation in a hemodynamically significant PDA would be that after closure, x-ray appearance should improve. The literature describes a phenomenon after closure of patients with either transient or progressive worsening oxygenation and ventilation. Following closure, there is an acute increase in systemic vascular resistance and simultaneous decrease in left ventricular preload, which can increase strain on the preterm left ventricle and upstream impact on the pulmonary vascular bed. When pulmonary venous pressure increases, this manifests as pulmonary edema (31). This phenomenon occurring would lead to a less impressive haziness change outcome overall. Another confounding factor is that many patients have significant lung disease, contributing to haziness on x-ray that is not solely edema. While distinguishing pulmonary edema from cystic emphysema or chronic lung disease is challenging, we aimed to attribute immediate postoperative changes to reduced pulmonary edema. Additionally, even though an attempt was made to blind the x-ray scorers from any patient identifiers and time progression during their CXR reading, we realize the presence of the PDA device on the CXR signifies a post-procedure CXR, which might have led to some bias.

Our study analyzed the clinical change in the immediate post-procedure period for our patients. Within a week of closure, 6% of patients required initiation of inotropes. This is similar with the incidence in another study, where they found 8% were diagnosed with low cardiac output syndrome (32). Although not addressed in this study, our institution has previously published that patients undergoing surgical ligation were more likely to require increased respiratory and vasoactive support postoperatively compared to infants undergoing TCDC (33). Our data showed that 40% of patients were able to wean respiratory support after TCDC. This result alone cannot be proven to be due to the procedure itself, as we know that patients that do not undergo intervention are still able to wean support with time. One study showed that the presence or prolonged duration of a PDA might not increase the rate of mortality or morbidities (34). However, we hypothesize that reduced pulmonary over-circulation during the immediate postoperative period also contributed to this weaning process. In 54% of the patients in this study, respiratory support was similar before and after the procedure. This datapoint is difficult to interpret in the absence of other variables. One proposed explanation is that patients who were more critically ill prior to the procedure may continue to require comparable levels of respiratory support postoperatively due to persistent critical illness and underlying lung pathology, despite successful PDA closure.

We then sought to analyze characteristics of patients to better understand which patients experienced more clinical benefit from TCDC, both in clinical respiratory status and by CXR appearance. Analysis was complicated due to the small counts seen in many of the patient categories. This caused instability in the analysis model results, which we attempted to mediate by combining some categories to increase counts in each. Early PDA closure in extremely low birth weight infants, particularly within the first 4 weeks of life, has been associated with preventing pulmonary vascular disease, faster somatic growth, shorter respiratory support duration, and earlier discharge compared to delayed closure (34–37). Although not statistically significant, our data did support trends seen in the literature, as the group of babies with significantly improved CXR's and improved respiratory status were more likely to be younger, weighed less than 2 kg, had more mild chronic lung disease, and had larger PDA's than the babies with less CXR improvement after TCDC. Considering that younger and smaller infants are known to have lung immaturity, one can theorize that pulmonary overcirculation from a hemodynamically significant PDA may lead to more pulmonary edema. The benefit from closure in these patients may be greater due to this varied effect. We did find that there was a statistically significant difference in those that had a higher CLD severity score, as patients with a higher score showed less benefit. The respiratory status in these patients changed less after intervention, likely because the PDA was not the main driver of their symptoms.

Our study leads us to agree with other institutions in how to determine which patients have a hemodynamically significant PDA. In our patient population, only the echo component of our modified McNamara score was associated with improvement, while the Total and Clinical score were not clearly associated. We propose that the clinical score may be falsely elevated in patients with other comorbidities, including CLD, and therefore explain why not as much benefit was achieved after intervention.

Our study was limited by its retrospective nature and our limited control over the timing and nature of the assessment points as well as the interventions. Even though we attempted to look at the relationship between immediate post-procedure radiographic and clinical changes within the first week after the intervention, we realize that our study was not powered to adequately assess the long-term outcomes or their determinants in our patient population. Additionally, given that our study was retrospective, data collection and interpretation were limited by reliance on accurate documentation in the medical record.

In conclusion, our study contributes to the growing evidence that transcatheter closure of the patent ductus arteriosus (TCDC) in premature infants, particularly those with lower birth weight and earlier intervention, may offer clinical benefits, including less evidence of pulmonary edema on CXR and improved respiratory support weaning. However, further studies are needed to better assess which neonates or clinical characteristics indicate more benefit from TCDC. Debates still exist about whether a hemodynamically significant PDA needs to be closed in the early neonatal period in these small neonates but can only be effectively assessed by a properly designed randomized control trial looking at those with and without intervention.

## Data Availability

The original contributions presented in the study are included in the article/Supplementary Material, further inquiries can be directed to the corresponding author.
