# Supplementary material for: Improvement of pulmonary edema and respiratory status after transcatheter PDA closure in the smallest and most premature infants
Source: Front Pediatr. 2025 Apr 24;13:1472431. doi: 10.3389/fped.2025.1472431 (PMC12058673; doi:10.3389/fped.2025.1472431)
Supplement: Supplementary file 1 [file Datasheet1.docx]

**Supplementary Information:**

| **Table A1: Summary of Patient Characteristics by D Therapy** | | | | | | | |
| --- | --- | --- | --- | --- | --- | --- | --- |
|  | | **Overall** | **Started/**  **Increased** **4(5.9%)** | **Not done/**  **Continued** **56(82.4%)** | **Decreased** **2(2.9%)** | **Discontinued** **6(8.8%)** | **p-value** |
| Extent of prematurity | Moderate-late + Very Preterm | 9 (13.2) | 1 (25.0) | 7 (12.5) | 0 (0.0) | 1 (16.7) | 0.5580 |
|  | Extremely Preterm | 59 (86.8) | 3 (75.0) | 49 (87.5) | 2 (100.0) | 5 (83.3) |  |
| Birth Weight Category | <1000 | 54 (79.4) | 4 (100.0) | 44 (78.6) | 2 (100.0) | 4 (66.7) | 0.7267 |
|  | 1000-2500 | 14 (20.6) | 0 (0.0) | 12 (21.4) | 0 (0.0) | 2 (33.3) |  |
| Weight at PDA Closure category | <1000 | 5 (7.4) | 0 (0.0) | 5 (8.9) | 0 (0.0) | 0 (0.0) | 0.9459 |
|  | 1000 - <2000 | 37 (54.4) | 3 (75.0) | 30 (53.6) | 1 (50.0) | 3 (50.0) |  |
|  | 2000 - <3000 | 15 (22.1) | 1 (25.0) | 12 (21.4) | 1 (50.0) | 1 (16.7) |  |
|  | 3000+ | 11 (16.2) | 0 (0.0) | 9 (16.1) | 0 (0.0) | 2 (33.3) |  |
| Age at PDA Closure (Months) | Mean ± SD | 1.9 ± 1.2 | 1.8 ± 0.5 | 1.9 ± 1.3 | 2.0 ± 0.0 | 1.8 ± 1.0 |  |
|  | Median (Min, Max) | 2.0 (1.0, 6.0) | 2.0 (1.0, 2.0) | 1.5 (1.0, 6.0) | 2.0 (2.0, 2.0) | 1.5 (1.0, 3.0) | 0.9184 |
| Surfactant Therapy (ST) | No | 7 (10.3) | 1 (25.0) | 6 (10.7) | 0 (0.0) | 0 (0.0) |  |
|  | Yes | 61 (89.7) | 3 (75.0) | 50 (89.3) | 2 (100.0) | 6 (100.0) | 0.5598 |
| Steroids | No Steroids | 43 (63.2) | 3 (75.0) | 35 (62.5) | 1 (50.0) | 4 (66.7) | 0.9530 |
|  | Decadron | 12 (17.6) | 0 (0.0) | 10 (17.9) | 1 (50.0) | 1 (16.7) |  |
|  | Inhalation/ Inhalation + Decadron | 13 (19.1) | 1 (25.0) | 11 (19.6) | 0 (0.0) | 1 (16.7) |  |
| CLD Diagnosis | I | 1 (1.5) | 0 (0.0) | 1 (1.8) | 0 (0.0) | 0 (0.0) | 0.2449 |
|  | II | 11 (16.2) | 2 (50.0) | 6 (10.7) | 1 (50.0) | 2 (33.3) |  |
|  | III | 11 (16.2) | 0 (0.0) | 11 (19.6) | 0 (0.0) | 0 (0.0) |  |
|  | NA* | 45 (66.2) | 2 (50.0) | 38 (67.9) | 1 (50.0) | 4 (66.7) |  |
| Indexed Size of PDA / BSA (mm/m2) | Mean ± SD | 23.3 ± 9.1 | 26.6 ± 2.8 | 23.4 ± 9.4 | 26.8 ± 6.9 | 19.3 ± 10.4 |  |
|  | Median (Min, Max) | 23.7 (4.3, 45.6) | 25.6 (24.5, 30.8) | 23.2 (4.3, 45.6) | 26.8 (21.9, 31.7) | 18.6 (6.8, 35.0) | 0.5323 |
| Echo McNamara Score | Mean ± SD | 4.3 ± 1.2 | 4.3 ± 1.0 | 4.3 ± 1.2 | 5.0 ± 0.0 | 4.3 ± 1.6 |  |
|  | Median (Min, Max) | 5.0 (1.0, 5.0) | 4.5 (3.0, 5.0) | 5.0 (1.0, 5.0) | 5.0 (5.0, 5.0) | 5.0 (1.0, 5.0) | 0.6794 |
| pHTN | No | 37 (54.4) | 2 (50.0) | 29 (51.8) | 1 (50.0) | 5 (83.3) |  |
|  | Yes | 31 (45.6) | 2 (50.0) | 27 (48.2) | 1 (50.0) | 1 (16.7) | 0.5731 |
| Total McNamara Score | Mean ± SD | 7.0 ± 1.2 | 6.8 ± 1.0 | 7.0 ± 1.3 | 7.5 ± 0.7 | 6.7 ± 1.4 |  |
|  | Median (Min, Max) | 7.0 (4.0, 9.0) | 6.5 (6.0, 8.0) | 7.0 (4.0, 9.0) | 7.5 (7.0, 8.0) | 7.0 (4.0, 8.0) | 0.7294 |
| Total McNamara Score >=7 | No | 18 (26.5) | 2 (50.0) | 15 (26.8) | 0 (0.0) | 1 (16.7) |  |
|  | Yes | 50 (73.5) | 2 (50.0) | 41 (73.2) | 2 (100.0) | 5 (83.3) | 0.6338 |

*NA not included in statistical comparison of CLD Diagnosis by D Therapy

| **Table A2: Summary of Characteristics by Support on Different Machine** | | | | | | |
| --- | --- | --- | --- | --- | --- | --- |
|  | | | **Change to Different Machine** | | | |
|  | | **overall** | **Increased Support** **1(3.0%)** | **No Change** **38(56.7%)** | **Decreased Support** **27(40.3%)** | **p-value** |
| Extent of prematurity | Moderate-late + Very Preterm | 9 (13.2) | 1 (50.0) | 4 (10.5) | 4 (14.8) | 0.2935 |
|  | Extremely Preterm | 59 (86.8) | 1 (50.0) | 34 (89.5) | 23 (85.2) |  |
| Birth Weight Category | <1000 | 54 (79.4) | 1 (50.0) | 32 (84.2) | 20 (74.1) | 0.2402 |
|  | 1000-2500 | 14 (20.6) | 1 (50.0) | 6 (15.8) | 7 (25.9) |  |
| Weight at PDA Closure category | <1000 | 5 (7.4) | 0 (0.0) | 2 (5.3) | 2 (7.4) | 0.7480 |
|  | 1000 - <2000 | 37 (54.4) | 1 (50.0) | 19 (50.0) | 17 (63.0) |  |
|  | 2000 - <3000 | 15 (22.1) | 1 (50.0) | 10 (26.3) | 4 (14.8) |  |
|  | 3000+ | 11 (16.2) | 0 (0.0) | 7 (18.4) | 4 (14.8) |  |
| Age at PDA Closure (Months) | Mean ± SD | 1.9 ± 1.2 | 1.5 ± 0.7 | 2.1 ± 1.2 | 1.8 ± 1.3 |  |
|  | Median (Min, Max) | 2.0 (1.0, 6.0) | 1.5 (1.0, 2.0) | 2.0 (1.0, 6.0) | 1.0 (1.0, 6.0) | 0.4306 |
| Surfactant Therapy (ST) | No | 7 (10.3) | 0 (0.0) | 3 (7.9) | 3 (11.1) |  |
|  | Yes | 61 (89.7) | 2 (100.0) | 35 (92.1) | 24 (88.9) | 0.7404 |
| Steroids | No Steroids | 43 (63.2) | 2 (100.0) | 23 (60.5) | 17 (63.0) | 0.3885 |
|  | Decadron | 12 (17.6) | 0 (0.0) | 5 (13.2) | 7 (25.9) |  |
|  | Inhalation/  Inhalation + Decadron | 13 (19.1) | 0 (0.0) | 10 (26.3) | 3 (11.1) |  |
| CLD Diagnosis | I | 1 (1.5) | 0 (0.0) | 1 (2.6) | 0 (0.0) | 0.2449 |
|  | II | 11 (16.2) | 0 (0.0) | 5 (13.2) | 6 (22.2) |  |
|  | III | 11 (16.2) | 0 (0.0) | 9 (23.7) | 2 (7.4) |  |
|  | NA* | 45 (66.2) | 2 (100.0) | 23 (60.5) | 19 (70.4) |  |
| Indexed Size of PDA / BSA (mm/m2) | Mean ± SD | 23.3 ± 9.1 | 28.9 ± 6.3 | 22.9 ± 9.5 | 22.9 ± 8.6 |  |
|  | Median (Min, Max) | 23.7 (4.3, 45.6) | 28.9 (24.4, 33.3) | 22.7 (4.3, 45.6) | 23.0 (6.8, 43.8) | 0.5955 |
| Echo McNamara Score | Mean ± SD | 4.3 ± 1.2 | 5.0 ± 0.0 | 4.2 ± 1.3 | 4.5 ± 1.0 |  |
|  | Median (Min, Max) | 5.0 (1.0, 5.0) | 5.0 (5.0, 5.0) | 5.0 (1.0, 5.0) | 5.0 (1.0, 5.0) | 0.3931 |
| pHTN | No | 37 (54.4) | 2 (100.0) | 19 (50.0) | 15 (55.6) |  |
|  | Yes | 31 (45.6) | 0 (0.0) | 19 (50.0) | 12 (44.4) | 0.5966 |
| Total McNamara Score | Mean ± SD | 7.0 ± 1.2 | 7.5 ± 0.7 | 6.9 ± 1.3 | 7.0 ± 1.1 |  |
|  | Median (Min, Max) | 7.0 (4.0, 9.0) | 7.5 (7.0, 8.0) | 7.0 (4.0, 9.0) | 7.0 (4.0, 9.0) | 0.8278 |
| Total McNamara Score >=7 | No | 18 (26.5) | 0 (0.0) | 11 (28.9) | 7 (25.9) |  |
|  | Yes | 50 (73.5) | 2 (100.0) | 27 (71.1) | 20 (74.1) | 0.9999 |

*NA not included in statistical comparison of CLD Diagnosis by Change to Different Machine

| **Table A3: Intra-rater Repeatability** | | | | | | | | |
| --- | --- | --- | --- | --- | --- | --- | --- | --- |
| **Rater** | **Variable** |  | **Mean ± SD** | **Median (Min, Max)** | **t-value** | **Degrees**  **of**  **Freedom** | **p-value** | **ICC** |
| A | Chest Xray 1 | 49 | 29.0 ± 11.3 | 32.0 (16.0, 48.0) |  |  |  |  |
|  | Chest Xray 2 | 49 | 31.2 ± 12.2 | 32.0 (16.0, 48.0) |  |  |  |  |
|  | Difference | 49 | -2.2 ± 8.2 | 0.0 (-18.0, 16.0) | -1.85 | 48 | 0.0710 | 0.99 |
| B | Chest Xray 1 | 49 | 29.2 ± 9.1 | 32.0 (13.0, 48.0) |  |  |  |  |
|  | Chest Xray 2 | 49 | 23.9 ± 10.4 | 24.0 (0.0, 48.0) |  |  |  |  |
|  | Difference | 49 | 5.2 ± 7.3 | 4.0 (-8.0, 16.0) | 5.01 | 48 | <.0001 | 0.61 |
| C | Chest Xray 1 | 49 | 29.2 ± 10.0 | 30.0 (13.0, 48.0) |  |  |  |  |
|  | Chest Xray 2 | 49 | 21.0 ± 13.5 | 17.0 (2.0, 48.0) |  |  |  |  |
|  | Difference | 49 | 8.1 ± 7.5 | 9.0 (-6.0, 29.0) | 7.64 | 48 | <.0001 | 0.62 |
| D | Chest Xray 1 | 49 | 31.9 ± 10.6 | 32.0 (8.0, 48.0) |  |  |  |  |
|  | Chest Xray 2 | 49 | 30.0 ± 11.2 | 32.0 (16.0, 48.0) |  |  |  |  |
|  | Difference | 49 | 1.9 ± 8.3 | 0.0 (-16.0, 16.0) | 1.58 | 48 | 0.1212 | 0.70 |

| **Table A4: Inter-rater Reliability** | | | | | |
| --- | --- | --- | --- | --- | --- |
| **Time** | **Rater** | **N** | **Mean ± SD** | **Median (Min, Max)** | **ICC** |
| Pre-Op CXR | A | 61 | 32.2 ± 12.0 | 32.0 (16.0, 48.0) | 0.72 |
|  | B | 61 | 33.0 ± 9.5 | 32.0 (16.0, 48.0) |  |
|  | C | 61 | 27.5 ± 11.9 | 26.0 (4.0, 48.0) |  |
|  | D | 61 | 33.9 ± 10.3 | 32.0 (12.0, 48.0) |  |
| Post-Op CXR 1 | A | 61 | 26.1 ± 9.4 | 32.0 (16.0, 48.0) | 0.72 |
|  | B | 61 | 26.0 ± 10.0 | 32.0 (0.0, 48.0) |  |
|  | C | 61 | 22.9 ± 12.0 | 21.0 (0.0, 48.0) |  |
|  | D | 61 | 28.6 ± 12.2 | 32.0 (4.0, 48.0) |  |
| Post-Op CXR 2 | A | 61 | 32.2 ± 12.0 | 32.0 (16.0, 48.0) | 0.72 |
|  | B | 61 | 33.0 ± 9.5 | 32.0 (16.0, 48.0) |  |
|  | C | 61 | 27.5 ± 11.9 | 26.0 (4.0, 48.0) |  |
|  | D | 61 | 33.9 ± 10.3 | 32.0 (12.0, 48.0) |  |
| Post-Op CXR 3 | A | 61 | 26.1 ± 9.4 | 32.0 (16.0, 48.0) | 0.72 |
|  | B | 61 | 26.0 ± 10.0 | 32.0 (0.0, 48.0) |  |
|  | C | 61 | 22.9 ± 12.0 | 21.0 (0.0, 48.0) |  |
|  | D | 61 | 28.6 ± 12.2 | 32.0 (4.0, 48.0) |  |
